# Rat subthalamic stimulation: Evaluating stimulation-induced dyskinesias, choosing stimulation currents and evaluating the anti-akinetic effect in the cylinder test

**DOI:** 10.1016/j.mex.2019.10.012

**Published:** 2019-10-14

**Authors:** Antti Huotarinen, Sakari Leino, Raimo K. Tuominen, Aki Laakso

**Affiliations:** a Helsinki University Hospital and Clinical Neurosciences, Neurosurgery, P.O. Box 266, Topeliuksenkatu 5, 00260, Helsinki, Finland; b Division of Pharmacology and Pharmacotherapy, Faculty of Pharmacy, University of Helsinki, P.O. Box 56, 00014, Helsinki, Finland

**Keywords:** Subthalamic high frequency stimulation, cylinder test, STN DBS, STN HFS, 6-OHDA, Parkinson’s disease, Cylinder test, Dyskinesia

## Abstract

In experimental deep brain stimulation of the subthalamic nucleus (STN HFS), stimulation currents just below the appearance threshold of stimulation-induced dyskinesias has often been used. The behavioral effect of STN HFS can be measured by the reversal of forelimb use asymmetry produced by hemiparkinsonism can be measured with the cylinder test among other tests. We used 18 Wistar rats with 6-hydroxydopamine induced hemiparkinsonism to test a customized scale to rate the severity of stimulation-induced dyskinesia; we then used these ratings to choose low and high stimulation currents. Subsequent cylinder tests showed that stimulation at the higher current, inducing mild and short-lived dyskinesias, was required for robust improvement in forelimb use, contradicting the use of currents below stimulation-induced dyskinesia threshold. It was also beneficial to separately count both all touches and first touches with the cylinder wall; this provided additional sensitivity and robustness to our results.

• Scoring stimulation-induced dyskinesias can be used as a quantitative measure of dyskinesias and to choose stimulation currents.
• Cylinder test scoring separately for both first and all touches can improve both sensitivity and reliability.
• STN HFS at a current producing short-lived dyskinesias was required for robust improvement in forelimb use asymmetry.

## Graphical abstract

**Figure:**
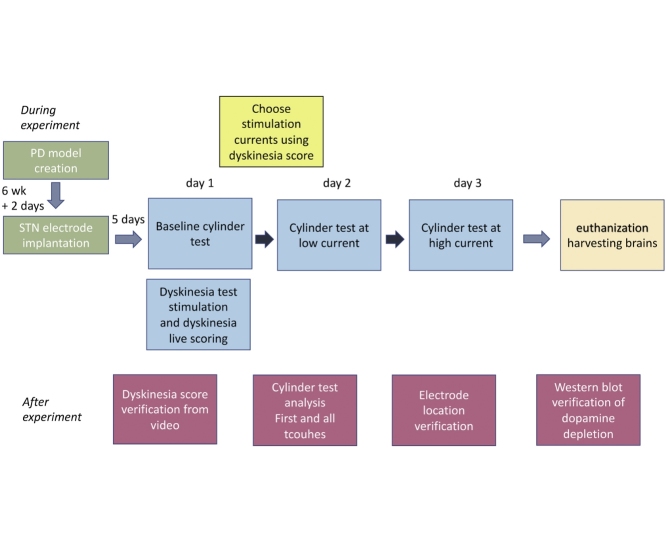

**Specification Table**

**Table:** 

Subject area:	*Neuroscience*
More specific subject area:	*Animal research of deep brain stimulation in Parkinson’s disease*
Method name:	*Subthalamic high frequency stimulation, cylinder test*
Name and reference of original method:	*Current selection based on stimulation-induced dyskinesias:* Salin P, Manrique C, Forni C, Kerkerian-Le Goff L. High-frequency stimulation of the subthalamic nucleus selectively reverses dopamine denervation-induced cellular defects in the output structures of the basal ganglia in the rat. J Neurosci. 2002;22(12):5137-5148. *Cylinder test forelimb use*: Schallert T, Fleming SM, Leasure JL, Tillerson JL, Bland ST. CNS plasticity and assessment of forelimb sensorimotor outcome in unilateral rat models of stroke, cortical ablation, parkinsonism and spinal cord injury. Neuropharmacology. 2000;39(5):777-787.
Resource availability:	*Videos available.*

## Method details

### Introduction

Deep brain stimulation (DBS) of the subthalamic nucleus (STN) is known to be an effective treatment for advanced Parkinson’s disease (PD), but its mechanisms remain elusive [[1], [2], [3]], warranting further animal studies. The neurotoxic rat unilateral 6-hydroxydopamine (6-OHDA) lesion model is one of the most important models used in PD research [4], including the study of STN DBS commonly termed high frequency stimulation in animal studies (STN HFS). STN HFS has been shown to effectively reverse the forelimb use asymmetry caused by a unilateral 6-hydroxydopamine (6-OHDA) injection [5,6], and also to reduce amphetamine-induced rotations {Fang:2010bw}, improve performance in stepping and Rotarod [7], and improve the speed of locomotion in the CatWalk test [8]. Dose responses under different stimulation amplitudes have been previously described for circling [7], dyskinesia thresholds [9], amphetamine-induced rotations, stepping tests, Rotarod tests, and premature responses [10], but not for the reversal of the contralateral forelimb akinesia in the cylinder test, which is one of the most commonly used behavioral tests in hemiparkinsonian rodents.

Previous literature has shown that STN DBS–induced dyskinesias predict clinically efficient stimulation [11]. In rodent STN HFS thresholds, different types of dyskinesias or automatic involuntary movements are induced [9]. A grading scale has been used to study the effects of STN HFS on L-DOPA–induced dyskinesias [12] but not for STN HFS-induced dyskinesias per se. STN HFS-induced dyskinesias have been rated mainly by the current where different subtypes of dyskinesias are induced [9]. Dyskinesias have also been previously used to determine individual stimulation amplitudes in rodent experiments by selecting a stimulation amplitude just below the threshold at which forelimb dyskinesias are induced [[13], [14], [15]]. Alternatively, a standard current can be used for all animals in the experiment [14,16]. In addition, stimulation-induced contralateral circling (i.e., locomotive dyskinesia) has been suggested as a behavioral criterion for successful rodent STN HFS [17]. However, several factors, including electrode design [14], anatomical accuracy, and the disease model used, can affect the range of suitable stimulation amplitudes [18,19].

Given the very small size of rodent STNs, there will inevitably be animals with anatomically suboptimal placement of stimulation electrodes, although high hit rates directly into rodent STNs have been reported [20]. In the clinical setting, there is still ongoing debate over the optimal stimulation site for STN DBS, and some experts place the optimal stimulation site outside the STN [21]. During stimulation, the electrical current spreads up to few hundred μm [22], and as the reversal of motor deficits is also expected outside the STN, effective stimulation can also be achieved with electrodes located outside the STN [23].

Given these considerable variations in the methodological details of rodent STN HFS, the aim of this study was: A) to further refine the method of grading STN HFS-–induced dyskinesias, as well as B) their use in choosing individual stimulation currents; C) to refine cylinder test behavioral analyses; D) to clarify the dose response of STN HFS in the reversal of forelimb use asymmetry; and E) to further explore the use of stimulation-induced dyskinesias as a marker of successful electrode placement in the STN.

## Materials and methods

### Animals and study design

We used male Wistar rats (n = 21; Harlan, Horst, The Netherlands) weighing 250–300 g at the beginning of the study. The rats were kept in a 12:12 h light/dark cycle with free access to rodent food (Harlan, Horst, the Netherlands) and tap water. All procedures were conducted during the light period. The rats were housed individually after the implantation of the stimulation electrodes. All animal experiments complied with ARRIVE guidelines and European Union Directive 2010/63/EU for animal experiments, and all procedures were approved by the Finnish National Animal Experiment Board (ESAVI-2010-07281).

**Fig. 1:**
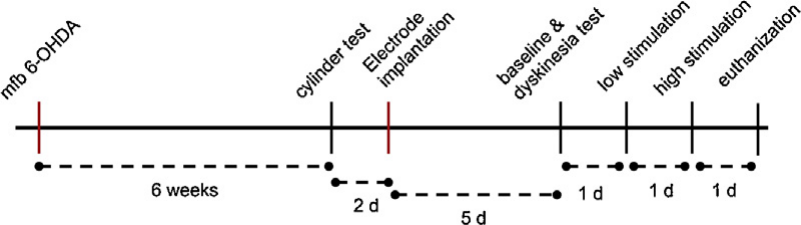
Experiment design. mfb = medial forebrain bundle, 6-OHDA = 6-hydroxydopamine.

An overview of the study design is presented in Fig. 1A and described below. First surgery was stereotactic surgery where 6-OHDA was injected to produce hemiparkinsonism. The behavioral effect of 6-OHDA was verified at six weeks after lesioning after which a second stereotactic operation was done to implant electrode the STN on the lesioned side. Five days after the second operation the actual testing of the behavioral effects of STN HFS took place on three consecutive days. On day one of the testing the rats first underwent baseline cylinder test to assess the degree of fore limb use asymmetry after which rats were stimulation at gradually increasing stimulation amplitudes to test for the dyskinetic effects of STN HFS. A low and high stimulation amplitudes were chosen based on these dyskinesia responses. On day two a new cylinder test was done using the low stimulation amplitude and on day three a cylinder test was done using the high stimulation amplitude. One rat was found dead in its cage before euthanasia. Additionally, two rats had incomplete datasets (one video recording failure and one case of badly cracked histological samples) and were excluded from further analysis.

### Surgery

Stereotactic procedures were conducted using a Stoelting rat stereotaxic frame (Wood Dale, IL, USA) while the rats were under isoflurane inhalation anesthesia (4.5% in the induction phase and 3–3.5% in the maintenance phase). Local anesthesia was applied subcutaneously, and 0.025 mg/kg of buprenorphine s.c. (Temgesic®, Indivior, Slough, UK) was administered for further analgesia. Prior to the 6-OHDA injections, desipramine (Sigma Chemical CO, St. Louis, MO, USA; 15 mg/kg, i.p. 1 ml/kg) was administered to prevent the uptake of 6-OHDA into the noradrenergic neurons. The rats received 8 μg of 6-OHDA (in 2 μl PBS, 0.1% ascorbic acid, kept on ice and shielded from light before injection) injected unilaterally into the medial forebrain bundle on the left side (L/M + 2.0, A/P −2.0, D/V −8.2). The behavioral effects of the lesion were tested at 6 weeks using a cylinder test. Two days after this, unilateral, concentric, bipolar, stainless steel stimulation electrodes, with a tip diameter of 150 μm and an outer electrode diameter of 460 μm with 1 mm between electrodes (MS 308/2, Plastics1, San Diego, CA, USA), were then implanted into the STN (L/M + 2.4, A/P −3.6, D/V −7.7 (from dura)) ipsilateral to the lesion and fixed to the skull. All coordinates are given relative to the bregma.

### Subthalamic stimulation and behavioral testing

After a five-day recovery period, a new cylinder test was performed with a cable and a swivel (SLC2C, Plastics1, San Diego, CA, USA) connecting the electrode to an impulse generator (STG 4004, Multichannel Systems, Reutlingen, Germany), allowing the rat to move freely. Video was recorded simultaneously from both beneath the cylinder and from its side. The cylinder test was performed on three consecutive days. On the first day, no stimulation was delivered for the first 10 min, after which the rats were stimulated with stepwise increasing currents (range: 0–400 μA, steps of 25 μA until 150 μA, then steps of 50 μA; frequency: 130 Hz; pulse width: 60 μs). The stimulation-induced dyskinesias were separately rated live on a scale from 0 to 4, adapted and modified from Cenci [24] especially to account for the transient nature [9] of stimulation-induced dyskinesias at lower amplitudes (0 = no dyskinesia; 1 = transient [<2 s], very mild; 2 = short [<10 s] and mild, or <50% of the time; 3 = marked [>10 s] and >50% of the time; 4 = extreme and persisting and >50% of the time). Dyskinesia was rated separately for orofacial, axial, forelimb, and locomotive dyskinesias at all tested stimulation currents. When maximal dyskinesia (defined as either excessive rotation endangering the implanted electrodes, or a rating of 4 on at least three of the subscales) was reached, the testing was stopped and the ratings from the highest current tested were used for the higher current data points. Using the videos, the dyskinesias were later rated independently by another researcher who was blinded to the initial dyskinesia ratings and cylinder test results (Video 1).

Based on the live dyskinesia ratings, two stimulation currents (low and high) were individually chosen for each animal. These currents were used in subsequent stimulation trials to study the effects of increasing stimulation currents and the relationship between stimulation-induced mild dyskinesias and their anti-akinetic effects. The low stimulation current was chosen to be at the current required for the onset of barely noticeable stimulation-induced forelimb dyskinesias. The high stimulation current was set at either just below the level at which stimulation-induced forelimb dyskinesias reached a rating of 3 on our adapted scale, or at the lowest threshold for a rating of 3, corresponding to clearly visible but short-lived forelimb dyskinesias. The low stimulation current was used on day two to study the anti-akinetic effect of a low current that produces only barely noticeable dyskinesias. The high stimulation amplitude was used on day three to study the anti-akinetic effect of a current that produced easily noticeable but mild and transient dyskinesias.

The dyskinesia subtype ratings from at each stimulation current were added together to form a dyskinesia score (DS) that reflected the amount of dyskinesia seen at the respective stimulation current amplitude. Overall sensitivity to stimulation-induced dyskinesia was measured by the sum of DS values across all stimulation amplitudes to form a total dyskinesia score (TDS). This was later used to divide rats into low and high dyskinesia groups.

The cylinder videos (excluding the dyskinesia testing video) were rated for forelimb use asymmetry by a researcher blinded to the dyskinesia ratings. During rearing movements, contacts made between the wall and the left or the right forepaw were counted using two different methods. In the first-touches method, only the first contact during each individual rearing was counted. In the all-touches method, all contacts with the wall were counted. Touches were counted as double (left and right paws) only if the contact was made in consecutive frames in a 29 fps video. All forelimb use statistical analysis was done using asymmetry, where each double touch was allocated evenly to the left and right forelimbs (100*(right + double)/(right+2*double + left)) (Video 2).

After the final stimulation, the animals were euthanized by decapitation. Their brains were dissected and snap-frozen in −40 °C precooled isopentane.

### Cresyl violet staining and western blot

Striata were harvested at −15 °C in a cryotome (CM3050 S, Leica, Wetzlar, Germany) with a 3 mm sampling punch; they were then stored at −80 °C. For western blotting (WB), the striata were homogenized and boiled for 10 min in 1% sodium dodecyl sulfate (SDS) and then sonicated. Their protein content was measured using DC protein assays (Biorad, Hercules, CA, USA) with a UV-2401PC spectrophotometer (Shimadzu, Kyoto, Japan) and a UV Probe 2.31 program (Shimadzu, Kyoto, Japan). Equal amounts of protein (20 μg) was then loaded onto 10% SDS gels (Biorad TGX), which were blotted with Biorad Turboblotter on PVDF membranes. Tyrosine hydroxylase was probed with an anti-tyorsine hydroxylase (TH) antibody (1:4000; #2792, Cell Signaling Technology, Danvers, MA, USA), visualized with Pierce ECL+ (ThermoFisher, Waltham, MA, USA),and detected with Typhoon 9000 chemifluorescence (GE Healthcare, Chicago, IL, USA). Loading control was performed by re-probing the same membrane with anti-actin (1:30000, Merck Millipore #MAB1501, Billerica, MA, USA). The signal intensity was quantified using ImageJ 1.49 (Mac version, NIH image, [25]), and the lesioned side was compared to the un-lesioned side.

Coronal sections of 40 μm were taken from the STN area in a cryotome. The sections were stained with 0.2% cresyl violet. They were then analyzed under a light microscope and compared to the Paxinos and Watson rat brain atlas [20].

### Statistical analysis

All statistical analysis was done using SPSS 24.0 software (IBM, Armonk, NY, USA). Cronbach’s alpha was used to study scale reliability, with values >0.9 indicating a high degree of consistency [26]. Lin’s concordance co-efficiency correlation was used to assess interrater reliability for dyskinesia ratings and scores, with values >0.9 indicating high reliability [27]. Either one- or two-way RM ANOVA tests were used, as appropriate. Bonferroni-corrected post-hoc tests were used to test for differences between cylinder tests at different stimulation currents. K-means cluster analysis was used to define a subpopulation of rats with a lower level of stimulation-induced dyskinesias using TDS. The correlation of forelimb use and stimulation-induced dyskinesias was tested with Spearman’s correlation. Linear fit equations (front limb use = a*dyskinesia score + b) were used to define DS values corresponding to a significant improvement of contralateral front limb use. P-values <0.05 were considered significant.

## Results

### Verification of the 6-OHDA lesion

**Fig. 2:**
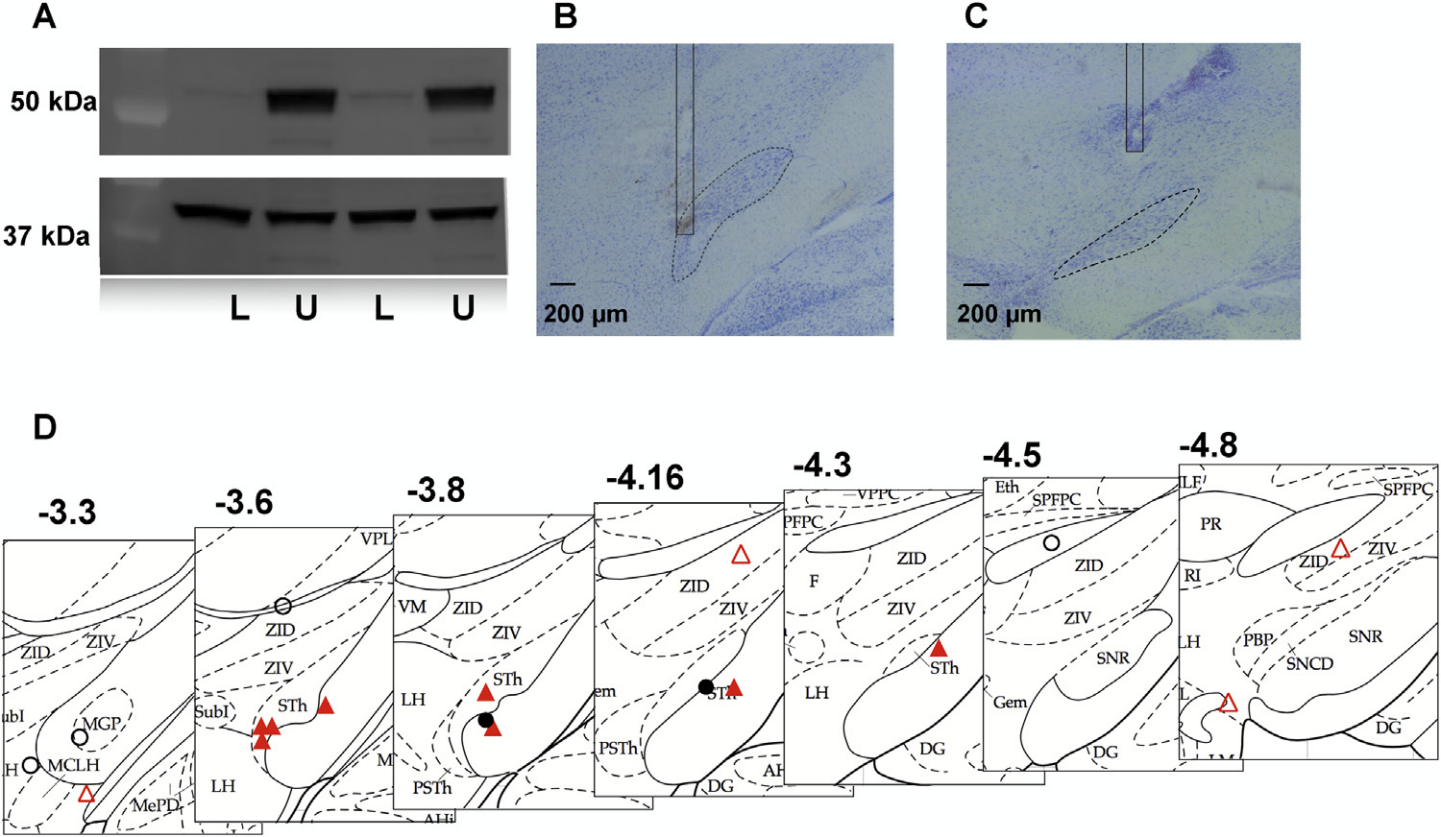
A) Biochemical verification of successful 6-OHDA lesioning, using western blot for tyrosine hydroxylase above; confirms dopamine depletion on the lesioned side. Results of actin loading control below. (L) compared to un-lesioned (U) striata. B) An example of good electrode placement within the STN. C) An example of missed electrode placement, despite a good observed anti-akinetic stimulation effect and high level of dyskinesia. D) Electrode locations in relation to Paxinos and Watson rat brain atlas images, with corresponding coronal sections (4th edition, Figures 33–39, used with permission from Elsevier). Anatomical hits (n = 10) within 200 μm of STN border are represented by solid markers and misses (n = 8) by hollow markers. Triangles represent rats with high dyskinesia response (n = 12) and circles represent rats with low dyskinesia response. L = lesioned side, U = unlesioned side, MGP = medial globus pallidus, MCLH = magnocellular nucleus of the lateral hypothalamus. STN/STh = subthalamic nucleus, ZI = zona incerta, dashed line = STN, solid line = electrode.

A dopaminergic lesion was present in all animals, as verified by WB, with a mean TH immunoreactivity of 13.8% (Fig. 2A) (95% CI [9.5–18,3%]) compared to the animal’s contralateral side. The rats showed preferential use of ipsilateral forelimbs in the cylinder test that was conducted before the electrode implantation (all touches 27.5%, 95% CI [21.5–33.9%]; first touches 10.2%, 95% CI [1.1–17.7%]).

### Location of the electrode tip

The tips of all stimulation electrodes were placed in the subthalamic area, with the mean electrode tip positions being A/P −3.90 (95% CI [−4.14 to −3.66]), M/L 2.66 (95% CI [1.95–3.37]) and D/V −7.75 (95% CI [−7.04 to −8.45]). Ten electrodes were either within ∼200 μm of the STN borders or inside the STN (Fig. 2B); these were classified as hits. Eight electrodes that were farther away (Fig. 2C) were classified as misses. There was no apparent difference in electrode location depending on the behavioral response (Fig. 2D).

### Stimulation-induced dyskinesias and scale reliability

The sum of the dyskinesia ratings on the different subscales (orofacial, axial, forelimb and locomotive) was tested with Cronbach’s alpha to produce a DS with high scale reliability for both live and video-based ratings (0.942 and 0.913, respectively). Removing orofacial dyskinesias slightly improved Cronbach’s alpha (0.953 and 0.950). The removal of any of the other subscales from the score worsened Cronbach’s alpha. In addition, orofacial dyskinesias proved to be difficult to grade from videos. Therefore, DS values that did not include orofacial dyskinesias were used for further analysis.

**Fig. 3:**
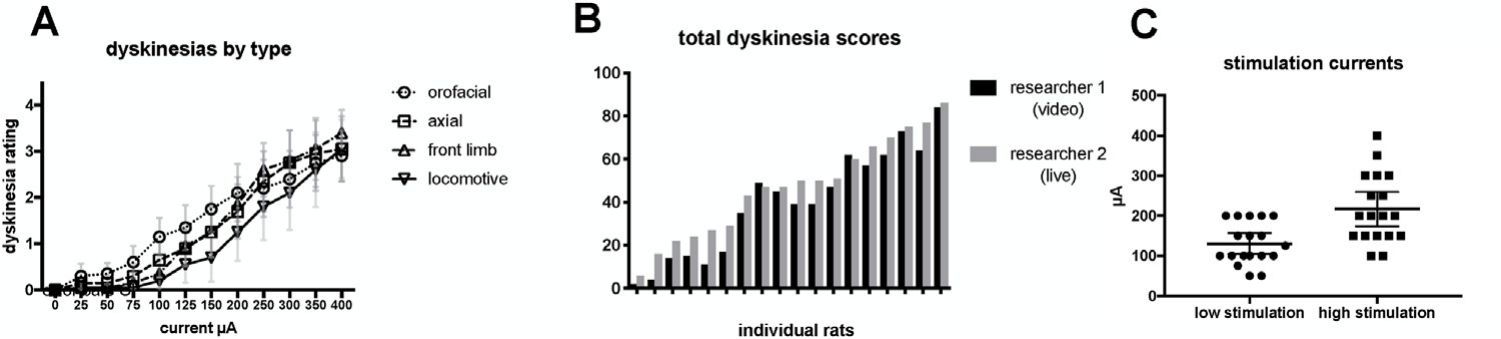
A) Stimulation-induced dyskinesias by dyskinesia type across all stimulation currents (n = 18). B) Sum of all dyskinesia ratings without the orofacial dyskinesias across all currents (total dyskinesia score, TDS, theoretical maximum 204). Each bar represents an individual rat ordered from the lowest to highest value, as rated from video (researcher 1) and live during stimulation (researcher 2). C) Amplitudes used for low and high stimulation. Each square or circle represents a single animal. Data presented as mean; error bars represent 95% CI.

All animals responded to stimulation with gradually increasing dyskinesias (DS different from zero at all currents >100 μA, p < 0.001). On average, the dyskinesias appeared in the following order: orofacial, axial, and forelimb, then locomotive (Fig. 3A). However, when summed over all tested currents (TDS), there was a large variation in the total amount of dyskinesias produced (Fig. 3B). The dyskinesia ratings given during live stimulation and those given by a blinded researcher during video playback were found to be highly concordant (>0.9) based on Lin’s concordance coefficient correlation [27].

Based on the live dyskinesia ratings, two stimulation currents were chosen for each rat and were used on the later cylinder tests (low stimulation 130 μA, 95% CI [104.4–156.7]; high stimulation 216.7 μA, 95% CI [174.0–259.4]; p < 0.001) (Fig. 3C).

### Cylinder test and reversal of forelimb use asymmetry

**Fig. 4:**
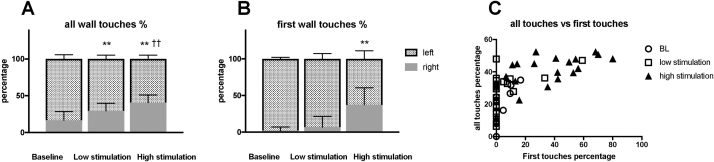
STN HFS, with both low and high stimulation, reduced forelimb use asymmetry in the cylinder test. A) The effect of STN HFS at two stimulation amplitudes on contralateral forelimb use was observed when counting all forepaw touches with the cylinder wall made during rearing movements. B) When counting only first forepaw touches with the wall during rearing movements, improvement in contralateral forelimb use was observed only with high stimulation. (A and B, stacked bar charts) C) Correlation was observed between measurements of contralateral forelimb use obtained by counting all touches (x-axis) and only first touches (y-axis) with the wall during rearing movements. Data presented as mean; error bars represent 95% CI. H = rats with high total dyskinesia score; L = rats with low total dyskinesia score. ** different from baseline: p < 0.01 Bonferroni-corrected post-hoc test. †† different from low stimulation: p < 0.01 Bonferroni-corrected post-hoc test.

In the cylinder test, all lesioned animals showed decreased forelimb use contralateral to the lesion (mean 13.5% of all touches, 95% CI [8.3–18.6%]; one-sample *t*-test: p < 0.0001). The rats reared 10, 17, and 20 times (median) during baseline, low stimulation, and high stimulation, respectively. During the rearing movements, the rats touched the wall 57, 69, and 112 times (median) during the respective cylinder tests, all analyses was done and data is presented using asymmetry of forelimb using as described above. Forelimb use improved with stimulation current amplitudes when measuring all touches (F [3,17] = 43.52, p < 0.0001) (Fig. 4A) and first touches (F [3,17] = 25.4, p < 0.0001) (Fig. 4B) with the wall. However, improvement with low stimulation current was seen only when the all-touches measurement was used (post-hoc p < 0.0001 for all touches and p = 0.76 for first touches). The asymmetry of forelimb use, measured by first and all touches with the wall, was correlated (Spearman’s r = 0.748, p < 0.0001) (Fig. 4C).

### Sensitivity to dyskinesia and its relation to anti-akinetic effects

**Fig. 5:**
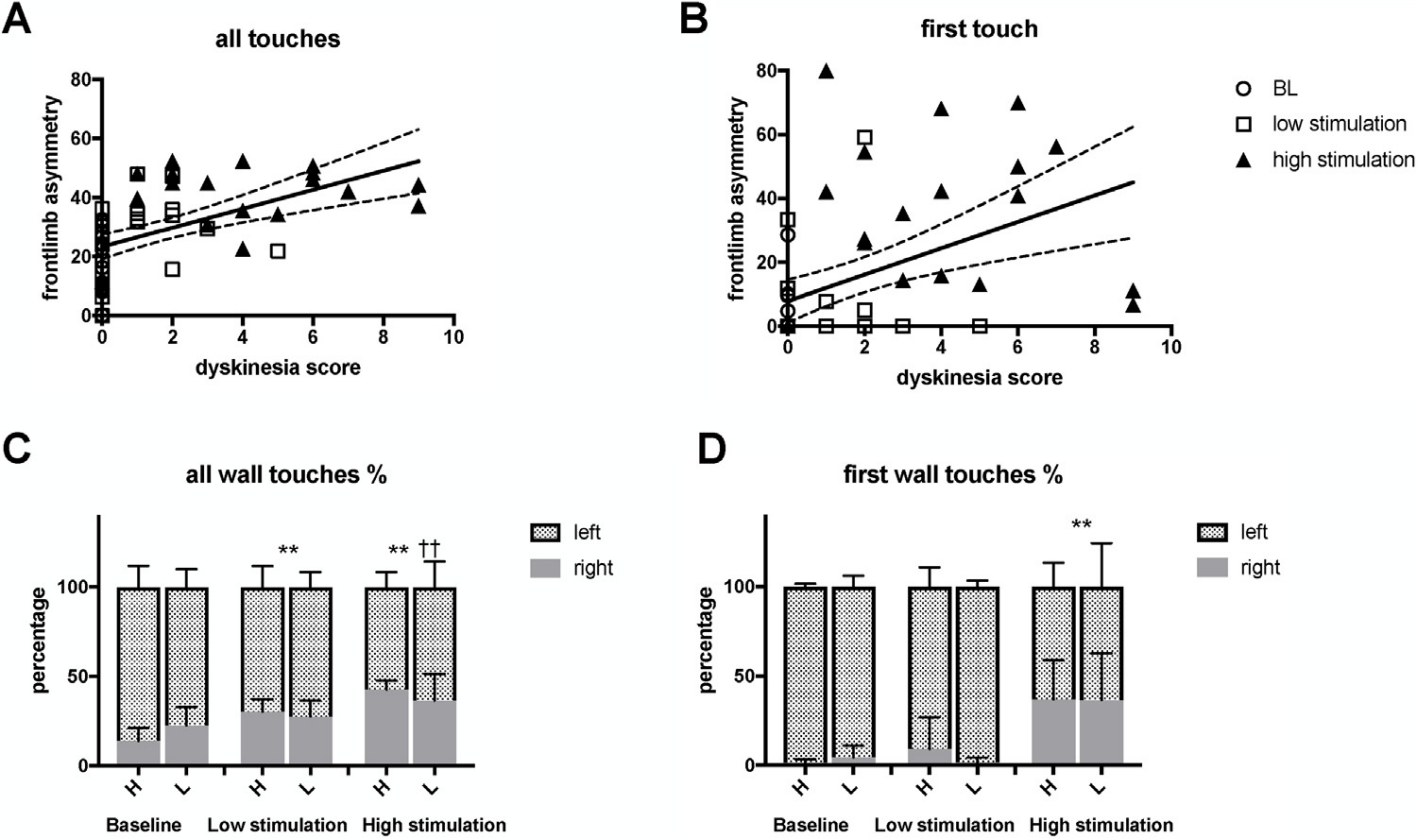
Contralateral forelimb use in the cylinder test correlated with dyskinesia ratings without orofacial dyskinesia. A) Contralateral forelimb use as measured by counting all touches with the cylinder wall during rearing movements. B) Contralateral forelimb use as measured by counting only first touches with the wall during rearing movements, compared to dyskinesia ratings observed at similar amplitudes during previous test stimulations. When rats were divided into low and high dyskinesia groups, there was no difference in forelimb use asymmetry in the cylinder test using either C) all touches or D) first touches, showing that similar efficacy of STN HFS was achieved by tailoring stimulation amplitude according to sensitivity to dyskinesia. (C and D, stacked bar charts). Solid line represents linear regression; dashed line represents 95% CI H = rats with high total dyskinesia score; L = rats with low total dyskinesia score.

To study the relation of stimulation-induced dyskinesias to the anti-akinetic effect, the correlation between dyskinesias and anti-akinetic effect was examined. Correlations were found between DS and forelimb use with both the all-touches (Spearman’s r = 0.683, p < 0.001) (Fig. 5A) and first-touches methods of assessment (Spearman’s r = 0.627, p < 0.001) (Fig. 5B).

Additionally, the animals were divided into low and high dyskinesia groups using K-means clustering of the TDS values, producing a low dyskinesia group (n = 6, TDS 17.8, 95% CI [2.3–16.4]) and a high dyskinesia group (n = 12, TDS 54.7, 95% CI [45.2–64.2]) whose anti-akinetic effects were then compared. Because the desired dyskinesia levels were reached with lower stimulation currents in the high dyskinesia group, the mean stimulation currents were lower for the high dyskinesia group than for the low dyskinesia group (low stimulation 103 μA, 95% CI [80–124] vs. 188 μA, 95% CI [155–220], *t*-test p = 0.002; high stimulation 167 μA, 95% CI [138–195] vs. 300 μA, 95% CI [243–357], *t*-test p < 0.001. Even when the improvements in the cylinder test controlled for the high and low dyskinesia groups, the effect of STN HFS was maintained using both the all-touches (two-way RM ANOVA F [2,15] = 26.4, p < 0.0001) (Fig. 5C) and first-touches (two-way RM ANOVA F [2,15] = 17.1, p = 0.0001) (Fig. 5D) assessments. However, there was no difference between the low and high dyskinesia groups using the all-touches (two-way RM ANOVA F [1,16] = 0.002, p = 0.97, all post-hoc tests >0.18) or first-touches (two-way RM ANOVA F [1,16] = 0.006, p = 0.94, all post-hoc tests >0.18) assessments in any of the stimulation conditions.

To determine the DS that corresponded to contralateral forelimb use of >40%, linear line fit equations for dyskinesia vs. forelimb use were solved for forelimb use asymmetry, as measured by counting all touches and first touches, resulting in 5.2 (95% CI [3.60–9.20]) for all touches (Fig. 5C) and 7.78 (95% CI [5.02–17.33]) for first touches (Fig. 5D).

### Electrode location and behavioral effect

**Fig. 6:**
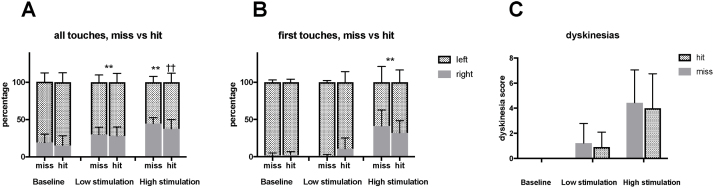
Comparison of forelimb use asymmetry between rats with good electrode placement and missed electrode placement in the cylinder test using A) all touches and B) first touches. (A and B, stacked bar charts) C) The sum of axial, forelimb, and locomotive dyskinesia ratings at low and high stimulation amplitudes, respectively. ** different from baseline: p < 0.01 Bonferroni-corrected post-hoc test. †† different from low stimulation: p < 0.01 Bonferroni-corrected post-hoc test.

There was no significant difference in the reversal of forelimb use asymmetry between electrodes placed inside the STN vs. those placed outside the STN but within the greater subthalamic area (Table 1 and Fig. 6A–B), as measured using both all touches (two-way RM ANOVA F [1,16] = 0.99, p = 0.34, all post-hoc tests >0.06) and first touches (two-way RM ANOVA F [1,16] = 0.008, p = 0.93, all post-hoc tests >0.18). TDS values were similar in rats with well-placed and missed electrodes (46.3, 95% CI [23.6–69.5], vs. 45.8, 95% CI [29.9–61.7]; *t*-test p = 0.96). Three rats showed less than 10% improvement in forelimb use asymmetry, but histological analysis showed that the electrode tip was placed within 200 μm of the STN (classified as a hit) in two of these cases.

**Table 1:** The results of behavioral testing, with a comparison between anatomical hits and misses.

			Anatomical miss (n = 8)	Anatomical hit (n = 10)	p-value
Use of contralateral front limb in cylinder test	All touches	BL	19.25	15.4	0.699
		low stimulation	30.1	28.04	0.187
		high stimulation	44.61	37.72	0.711
	First touches only	BL	1.84	2.62	0.747
		low stimulation	0.96	10.92	0.151
		high stimulation	41.52	32.17	0.422

Dyskinesia score	BL		0	0	–
	low stimulation		1.85	1.71	0.161
	high stimulation		6.38	5.8	0.711

## Discussion and conclusions

We studied the dyskinetic and anti-akinetic effects of STN HFS and their relations to electrode location in the rat 6-OHDA model of PD to further refine select methodological issues.

We found that dyskinetic effects could be graded live during test stimulations with gradually increasing stimulation currents. The dyskinesia rating was tailored from pharmacology experiments [24] to better account for the transient nature of STN stimulation-induced dyskinesias. However, orofacial dyskinesias proved to be nearly impossible to rate, especially from video recordings and with higher stimulation amplitudes. Grading dyskinesias in this fashion can nonetheless provide for greater fidelity than in the commonly used method of reporting threshold where dyskinesia appears [9]. This can then be used to choose lower or higher stimulation currents to provide for both modest and considerable improvements in akinesia on individual bases. This is particularly important since we observed considerable individual variation in the currents that induced the desired levels of dyskinesias and anti-akinetic effect, although the range was within what has been previously used for stainless steel electrodes [9,10,14]. Overall, our data supports the use of individually chosen stimulation currents, rather than a standard current for all animals, to achieve more reliable reversal of akinesia. Additionally, using dyskinesia ratings permits for quantitative follow-up during repeated testing. This may be especially useful if additional treatments are co-delivered with STN HFS.

Our data showed that stimulation with a lower current that produced only barely noticeable dyskinesias produced very little improvement in forelimb use asymmetry, so the common level of stimulation just below the threshold for dyskinesias would likely have been even less effective. The gradual increase of dyskinesias challenges the previously assumed validity of the appearance of dyskinesias threshold used to select individual stimulation parameters [9,12]. Even in animals with very low sensitivity to stimulation-induced dyskinesias, an anti-akinetic effect was achieved using higher stimulation currents. These findings suggest that instead of a dyskinesia appearance threshold, a rating scale should be used to select individual stimulation currents in rodent experiments, to better account for the relationship between anti-akinetic effect and dyskinesia and for the gradually increasing nature of stimulation-induced dyskinesias.

In this study, we achieved a reversal of forelimb use asymmetry, corresponding to good therapeutic effects with stimulation currents that corresponded to DS 4 or 5. This in turn corresponds to transient and mild dyskinesias that could no longer be observed after a few seconds. We also saw some hindlimb dyskinesias and rare ipsilateral dyskinesias, perhaps reflecting the somatotopy of STN [28]. In clinical DBS, an optimal therapeutic balance is sought between side-effects, such as dyskinesias, and the reduction of motor symptoms [29]. On the other hand, clinical DBS stimulation can be increased more gradually than in animal experiments, which allows for better adaptation to side-effects, which might in turn explain the need to use dyskinesia-eliciting currents in rodent DBS when currents can be introduced faster. Furthermore, in animal experiments, STN HFS is usually the only form of anti-akinetic treatment, contradicting clinical STN DBS in which L-DOPA is concurrently used, although a small minority of patients can transiently stop L-DOPA after STN DBS [30]. Thus, in animal studies, higher currents must be used with STN HFS to achieve a robust reduction of akinesia.

Forelimb use measured by both first and all touches with the wall during the cylinder test rearing movements were strongly correlated but showed different results. When only the first touch was counted, a reversal of forelimb use asymmetry was seen only with the higher stimulation current, which suggests that this is a more robust effect than that of the all-touches metric. The small improvement with the lower current was seen only when all touches were counted, and this method also revealed a previously unreported response to different stimulation amplitudes effect in the cylinder test. This may suggest that first touch measure reflects a more complex movement as during first touches more weight seems to be put on that paw. Conversely, all touches may provide a measure for less complex movement or reversal of akinesia as the there seems to be less weight bearing during these touches. In addition, analyzing all touches with the wall increased the total number of observed touches, thereby improving statistical power, which may may be more important especially for studies where the effect of STN HFS is followed over repeated measures as during repeated cylinder test the rats tend to explore less leading to smaller number of wall touches, as is shown also by our data. However, the neurophysiologic mechanism underlying this difference cannot be deciphered from our data and it warrants further studies. We therefore suggest that both these methods of rating cylinder tests should be used rather than just one or the other, as has previously been done [6,31].

Furthermore, a therapeutic anti-akinetic effect was found not only with electrodes implanted in the STN but also with electrodes implanted in the larger subthalamic area, including the zona incerta dorsal to the STN, as has been reported in clinical DBS [23,32]. It is also necessary to note that because the electrode configuration used in this study had a thinner active electrode in the tip and 1 mm proximal and superior to it a larger electrode (current sink), the electric field practically always spans over the zona incerta and fibers traversing to and from the STN [33]. This could suggest that using a good therapeutic response, instead of a good anatomical hit, as the major inclusion criterion in rat studies might better simulate clinical DBS, depending on the research question. Moreover, we found that the degree of stimulation-induced dyskinesias was similar for rats with hits and misses. It is plausible that this discrepancy could be explained by missing the electrode tip in the histological analysis or by the electrode tip shifting during removal and creating a misleading artifact in the histological analysis. However, this would suggest that animals with slightly displaced electrodes in histologic analyses should not necessarily be excluded if their behavioral response is good.

One of the limitations of the present study is that it used only one test for akinesia, the cylinder test, which does not account for skilled movement [35], locomotor activity [36], balance [37], or axial preference [38]. Additionally, using a fixed table-top impulse generator that requires connecting wires and swivels might hinder or even prevent using some of these tests. This could be overcome by the use of small impulse generators requiring no connecting wires or swivels [34]. However, the dose response was seen with similar currents in the stepping test, Rotarod, and amphetamine-induced rotations in previous studies [7]. Similarly, the short time-span and episodic nature of the stimulation might affect the dyskinetic and anti-akinetic range seen in this study. Further research is needed to show if similar relationships hold between other behavior tests and stimulation-induced dyskinesias. Additionally, electrophysiological measurements, such as microelectrode monitoring [39] or cortical EEG for beta rhythm monitoring [35], were not used.

In conclusion, dyskinesia ratings using a specifically tailored method may be suitable for grading STN HFS–induced dyskinesias, and this method can be used to define stimulation currents for experimental STN HFS. Cylinder test analyses can be improved by counting both first and all touches when scoring, and dose response can be particularly well observed when using all touches for scoring. In addition, stimulation-induced dyskinesias seemed to predict behaviorally effective STN HFS, but exact electrode placement inside the STN did not.

SL and RKT report no potential conflicts of interest.

AH received funding for work from the Finnish Government (grants for academic healthcare research) and the Finnish Parkinson Foundation. Conference fees and travel expenses have been paid by Medtronic, St. Jude and AbbVie.

## Declaration of Competing Interest

AH received funding for work from the Finnish Government (grants for academic healthcare research) and the Finnish Parkinson Foundation. Conference fees and travel expenses have been paid by Medtronic, St. Jude and AbbVie.
